# Proteomic Characterization of Human Neural Stem Cells and Their Secretome During *in vitro* Differentiation

**DOI:** 10.3389/fncel.2020.612560

**Published:** 2021-01-28

**Authors:** Jakub Červenka, Jiřina Tylečková, Helena Kupcová Skalníková, Kateřina Vodičková Kepková, Ievgeniia Poliakh, Ivona Valeková, Lucie Pfeiferová, Michal Kolář, Michaela Vaškovičová, Tereza Pánková, Petr Vodička

**Affiliations:** ^1^Laboratory of Applied Proteome Analyses, Research Center PIGMOD, Institute of Animal Physiology and Genetics of the Czech Academy of Sciences, Liběchov, Czechia; ^2^Department of Cell Biology, Faculty of Science, Charles University, Prague, Czechia; ^3^Laboratory of Cell Regeneration and Plasticity, Research Center PIGMOD, Institute of Animal Physiology and Genetics of the Czech Academy of Sciences, Liběchov, Czechia; ^4^Laboratory of Genomics and Bioinformatics, Institute of Molecular Genetics of the Czech Academy of Sciences, Prague, Czechia; ^5^Department of Informatics and Chemistry, Faculty of Chemical Technology, University of Chemistry and Technology, Prague, Czechia; ^6^Laboratory of DNA Integrity, Research Center PIGMOD, Institute of Animal Physiology and Genetics of the Czech Academy of Sciences, Liběchov, Czechia

**Keywords:** neural stem cell, proliferation, neural differentiation, secretome, proteome, VEGF, SWATH-MS

## Abstract

Cell therapies represent a promising approach to slow down the progression of currently untreatable neurodegenerative diseases (e.g., Alzheimer's and Parkinson's disease or amyotrophic lateral sclerosis), as well as to support the reconstruction of functional neural circuits after spinal cord injuries. In such therapies, the grafted cells could either functionally integrate into the damaged tissue, partially replacing dead or damaged cells, modulate inflammatory reaction, reduce tissue damage, or support neuronal survival by secretion of cytokines, growth, and trophic factors. Comprehensive characterization of cells and their proliferative potential, differentiation status, and population purity before transplantation is crucial to preventing safety risks, e.g., a tumorous growth due to the proliferation of undifferentiated stem cells. We characterized changes in the proteome and secretome of human neural stem cells (NSCs) during their spontaneous (EGF/FGF2 withdrawal) differentiation and differentiation with trophic support by BDNF/GDNF supplementation. We used LC-MS/MS in SWATH-MS mode for global cellular proteome profiling and quantified almost three thousand cellular proteins. Our analysis identified substantial protein differences in the early stages of NSC differentiation with more than a third of all the proteins regulated (including known neuronal and NSC multipotency markers) and revealed that the BDNF/GDNF support affected more the later stages of the NSC differentiation. Among the pathways identified as activated during both spontaneous and BDNF/GDNF differentiation were the HIF-1 signaling pathway, Wnt signaling pathway, and VEGF signaling pathway. Our follow-up secretome analysis using Luminex multiplex immunoassay revealed significant changes in the secretion of VEGF and IL-6 during NSC differentiation. Our results further demonstrated an increased expression of neuropilin-1 as well as catenin β-1, both known to participate in the regulation of VEGF signaling, and showed that VEGF-A isoform 121 (VEGF121), in particular, induces proliferation and supports survival of differentiating cells.

## Introduction

The neural stem cells (NSCs) are undifferentiated cells capable of reproducing themselves and giving rise to progenitors that may further differentiate into neuronal and glial (astrocyte and oligodendrocyte) lineages. Establishment of techniques to isolate, *in vitro* propagate, and differentiate stem/progenitor cells from the fetal and adult central nervous system (CNS) and, more recently, to derive such NSCs or neural progenitor cells (NPCs) from embryonic stem cells (ESCs) or induced pluripotent stem cells (iPSCs) opened new avenues toward our understanding of nervous system development, enabled modeling of selected diseases, neurotoxicity, drug testing, and importantly, opened the research of regenerative potential of such cells [see Gage and Temple (2013) for review].

NSCs/NPCs are among cell sources considered or already used in clinical trials for cell-based therapies in neurological conditions including neurodegenerative diseases, e.g., Alzheimer's disease (Oliver and Reddy, 2019; Hayashi et al., 2020; Liu et al., 2020), Parkinson's disease (Díaz, 2019; Oliver and Reddy, 2019; Harris et al., 2020), amyotrophic lateral sclerosis (Abati et al., 2019), spinal cord injuries (Ahuja et al., 2020), stroke (Suda et al., 2020), or multiple sclerosis (Cuascut and Hutton, 2019; Pluchino et al., 2020). Currently, 40 studies employing NSCs/NPCs are Active or Completed according to ClinicalTrials.gov.

The transplanted cells are expected to influence local microenvironment, reduce cellular stress *in situ*, promote cell survival, modulate inflammation, enhance remyelination, and maintain neuronal circuits (de Gioia et al., 2020; Fischer et al., 2020; Ottoboni et al., 2020). Some of these effects are mediated by a direct cell-to-cell contact, others by release of simple metabolites. However, a significant part of the effects is expected to come from secreted proteins including cytokines, growth factors, and other proteins essential for cellular communication and signaling (Kupcova Skalnikova, 2013), leading to, e.g., anti-inflammatory effects (Einstein et al., 2007) or inhibition of apoptosis (Petrenko et al., 2020).

Vascular endothelial growth factor A (VEGF-A) is essential for CNS vascularization and development, as its depletion leads to reduced vascularity and consequently to decreased neuronal proliferation and increased neuronal death in mice (Haigh et al., 2003). Depletion of VEGF-A by inactivation of one of its alleles causes failure of vasculogenesis and embryonal lethality in mouse, suggesting very strict regulation of VEGF-A abundance (Carmeliet et al., 1996; Ferrara et al., 1996). In mammals, the VEGF protein family is represented by five factors: VEGF-A, VEGF-B, VEGF-C, VEGF-D, and placenta growth factor (PlGF), all acting as homodimers *in vivo* (Koch and Claesson-Welsh, 2012). Tischer et al. (1991) provided evidence that three different protein isoforms can be produced from the VEGF-A gene through alternative exon splicing, which are composed of 121 (VEGF121), 165 (VEGF165), or 189 (VEGF189) amino acid residues in human. In the last decade, evidence of VEGF-A pleiotropic functions in the nervous system was uncovered (see Mackenzie and Ruhrberg (2012) for review].

Characterization of stem/progenitor cell populations on the molecular level helps us to understand key processes involved in cell proliferation and differentiation and also identify markers of cells on certain differentiation stages or lineage commitment for potential sorting of highly purified cell populations (Zizkova et al., 2015). Current proteomic techniques based on liquid chromatography with tandem mass spectrometry (LC-MS/MS) can identify and quantify thousands of proteins in biological samples (Shoemaker and Kornblum, 2016). In addition, antibody-based techniques allow highly sensitive quantification of low abundant proteins, such as secreted growth and trophic or chemotactic factors (Skalnikova et al., 2011; Valekova et al., 2015). Using such techniques, we have previously characterized changes in cellular and signaling proteins during differentiation of porcine NSCs (Skalnikova et al., 2007, 2008) and in cell surface N-glycoproteins of human NSCs (Tyleckova et al., 2016).

In this study, we performed quantitative proteomic analysis of *in vitro* cultured human NSCs derived from the H9 ESC line. We have compared cellular and secreted protein levels between proliferating NSCs and cells differentiated for 7, 14, 21, and 28 days. Two differentiation protocols were compared, i.e., spontaneous differentiation by withdrawal of growth factors (epidermal growth factor, EGF; fibroblast growth factor 2, FGF2) and differentiation by withdrawal of EGF/FGF2 with trophic support by brain-derived neurotrophic factor (BDNF) and glial cell line-derived neurotrophic factor (GDNF) supplementation. Our results extend the knowledge of the regulatory networks behind NSC proliferation, survival, migration, and differentiation. Elucidation of these molecular mechanisms helps us to understand the stem cell behavior in nervous system development, tissue regeneration, and cancer.

## Materials and Methods

### Cell Culture

Unless stated otherwise, cell culture reagents were obtained from Life Technologies (Thermo Fisher Scientific). Cell cultures were maintained at 37°C in 5% CO_2_ in a humidified atmosphere.

#### Neural Stem Cells (NSCs)

Human NSCs (Thermo Fisher Scientific) derived from the NIH-approved H9 (WA09) human ESCs were cultivated according to the supplier's instructions. Briefly, NSCs were incubated on plates coated with 20 μg/mL poly-L-ornithine and 5 μg/mL laminin (both Sigma-Aldrich) in the basal NSC medium comprising KnockOut Dulbecco's modified Eagle's medium (DMEM)/F-12, 2 mM GlutaMAX, 1% penicillin–streptomycin, and 2% StemPro Neural Supplement and supplemented with 20 ng/mL human recombinant EGF (PeproTech) and 20 ng/mL human recombinant FGF2 (PeproTech) (proliferation medium). The medium was changed completely every other day, and cells were passaged every 3–5 days using Accutase (Sigma-Aldrich) or 0.05% trypsin/ethylenediaminetetraacetic acid (EDTA).

#### NSC Differentiations

NSCs were grown in the proliferation medium until they reached ~70% confluency. To induce spontaneous (S) differentiation by growth factor withdrawal, the medium was switched to the basal NSC medium. For spontaneous differentiation with trophic support (BG differentiation), cells were cultured in basal NSC medium supplemented by 10 ng/mL human recombinant BDNF and 10 ng/mL human recombinant GDNF (both PeproTech). During the differentiation, half of the medium was changed every other day. Cells in four replicates for MS analyses or in three replicates for immunoblotting and RT-qPCR were detached manually at day 0 (proliferating NSCs) and at days 7, 14, 21, and 28 during both NSC differentiations.

#### VEGF Induction

To analyze the influence of VEGF on NSC proliferation and survival, NSCs were cultivated in an IncuCyte FLR (Essen BioScience Inc.) incubator microscope. Cells were seeded into 24-well plates in either NSC proliferation medium or NSC basal medium. Both media were either left as is (control) or were further supplemented with 100 ng/mL human VEGF121, 100 ng/mL human VEGF165 (both PeproTech; 100–20A and 100–20), or their combination. Half of the medium was changed every other day. Cell growth was followed for 8 days, with 9 images acquired per well in hourly intervals and six replicates (wells) per condition. Data were analyzed for confluence in IncuCyte 2010A Rev2 software. The experiment was repeated three times from independent NSC cultivations.

#### Cell Viability Analysis

Cell viability was assessed by ReadyProbes Cell Viability Imaging Kit (Thermo Fisher Scientific) according to the manufacturer's instructions after 8 days of cultivation (VEGF induction, see above) within three bioreplicates and three scans per bioreplicate. The experiment was repeated two times. The image analysis was done in Fiji, version 1.52n (Schindelin et al., 2012). Images were segmented by Auto thresholding plugin using the Otsu method (Otsu, 1979). The number of Hoechst 33342-positive and SYTOX Green-positive cells was measured by 3D Object Counter plugin (Bolte and Cordelières, 2006).

### Sample Preparation for Secretome and MS Analyses

A cell culture medium (4 biological replicates, i.e., 4 culture dishes per condition) conditioned for 2 days was collected at days 0, 7, 14, 21, and 28 of both differentiation protocols, centrifuged (1,000 g at 4°C for 5 min), and filtered through a 0.22-μm syringe filter to remove cells and debris. Inhibitors of proteases (Roche) were added, and the medium was kept at −80°C until secretome analysis.

Cells on culture dishes used for medium collection were washed with PBS and harvested mechanically. Cells were then lysed with 8 M urea and 5 mM EDTA in 50 mM ammonium bicarbonate and homogenized by MultiSpin (Grant Instruments Ltd.) for 10 cycles, 1 min at 6,000 RPM, 20 s hard, and sonicated for 15 min in an ice-cold sonication bath (Bandelin electronic GmbH & Co. KG) with ultrasonic frequency 35 kHz. The protein concentration was determined by Pierce 660 nm protein assay (Thermo Fisher Scientific). Each cell lysate sample was then treated with ProteaseMAX surfactant (Promega) to a final concentration of 0.1% (w/v), reduced with tris(2-carboxyethyl)phosphine hydrochloride to a final concentration of 10 mM for 30 min at 32°C and alkylated with iodoacetamide to a final concentration of 40 mM for 45 min at room temperature in the dark. Samples were diluted with 50 mM ammonium bicarbonate to a final concentration of 1 M urea and 0.02% ProteaseMAX, followed by enzymatic digestion with endoproteinase LysC for 2 h at 37°C and trypsin overnight at 37°C, both at a 1:100 enzyme:substrate ratio. The digestion was stopped with formic acid (FA) at a final concentration of 2%. The resulting peptide mixtures were centrifuged at 20,000 g for 15 min at 4°C, and the supernatants were desalted on C18 spin columns (MacroSpin or MicroSpin columns, Nestgroup). The size of the spin column was selected based on the initial protein sample load. The eluted peptides were vacuum centrifuged to dryness and resuspended in aqueous solution with 2% acetonitrile (ACN) and 0.5% FA. The peptide concentration was determined from absorbance at 280 nm (Synergy HTX, BioTek). The peptide samples were diluted to a final concentration of 0.5 μg/μL with 1:30 (v/v) of spiked-in indexed Retention Time (iRT) peptides (Biognosys AG). The pooled sample was prepared as a mix of randomly chosen peptide samples from S and BG differentiation (from day 7 to 28), one sample per time point and differentiation (4 to 9 μg of each sample). The pooled sample was measured in between the other samples as a quality control and was also used for library development.

### LC-MS/MS Analyses

Individual samples were analyzed in randomized order. The peptide mixtures in 2% ACN in 0.5% FA were loaded for 10 min at 2 μL/min on Acclaim PepMap 100 C18 (5 μm, 0.1 × 20 mm; Thermo Fisher Scientific) trap column. The separation was performed on an in-house-packed 25-cm fused-silica column (75-μm inner diameter) with ProntoSIL 120 Å 3 μm C18 AQ beads (Bischoff Analysentechnik GmbH) in a trap-elute mode, using the Eksigent nano-LC 425 (Sciex) on-line connected to 5600+ TripleTOF (Sciex). A linear gradient was set to 5–35% ACN in 0.1% FA over 120 min and 35–50% ACN in 0.1% FA over 10 min at a flow rate of 200 nL/min. For data-dependent acquisition (DDA) mode, the top 30 precursors with accumulation time 300 ms and mass range 400–1,250 in high-sensitivity mode were fragmented (MS/MS accumulation time 150 ms and mass range 170–1,500 Da) in each cycle (exclusion time 13 s). For Sequential Window Acquisition of All Theoretical Mass Spectra (SWATH-MS), 35 variable windows calculated with a SWATH Variable window calculator (Sciex) were monitored with 150 ms accumulation time in MS and 100 ms accumulation time in MS/MS with mass ranges of 400–1,250 Da and 170–2,000 Da, respectively, and a cycle time of 3.5 s.

### MS Data Processing

The MS/MS spectra from DDA for each condition (NSCs, S7, S14, S21, S28, BG7, BG14, BG21, BG28) were merged and processed in Mascot Distiller 2.7.1 (Matrix Science Ltd.) and searched for protein identification and data export for spectral library building in Mascot Server 2.6.2 (Matrix Science Ltd.), using the Swiss-Prot human database (version from 6.2.2018, 20,245 proteins) with a list of common contaminants and β-galactosidase from *Escherichia coli*. Mass tolerances of peptides and fragments were set to 20 ppm, carbamidomethylation of cysteine was set as a fixed modification, and protein N-terminal acetylation and methionine oxidation were set as variable modifications. One missed cleavage was allowed, and specificity of digestion was set as cleavage after arginine, unless proline proceed, and always cleavage after lysine. The peptide-centric SWATH-MS data analysis was performed in Skyline-daily (version 4.1.1.18179) (MacLean et al., 2010). The sample-specific spectral library was built from 37 DDA runs. At least three samples were measured in DDA mode per time point and condition for sample library development including four pool sample measurements for library enrichment. The library cutoff score was set to 0.99. Ion mass tolerance was 0.05 m/z. Peptide retention times were calibrated and aligned between individual runs using the iRT peptides as standards. Raw SWATH-MS data were extracted in high-selectivity extraction mode with 23,000 resolving power. mProphet (Reiter et al., 2011) was used for peak picking in a range of scans within 10 min of predicted retention time. Decoy peptides for the mProphet model were generated with a shuffle sequence method. Repeated and duplicated peptides were removed from the dataset. A minimum number of two peptides (each with three transitions) per protein were used for final data export. The quantitative information for each transition (transition intensity defined as an area under the curve, information about retention time, and detection q-value) was exported from Skyline and further processed in MSstats R package (Choi et al., 2014) to obtain individual protein abundances. The iRT peptides, peptides with oxidized methionine, and peptides with *q* > 0.01 were removed from the data using SkylinetoMSstats function. For between-run normalization, all intensity values were scaled by a factor calculated as global median total ion current (TIC) divided by run-specific median TIC. Protein abundances for each run were calculated from log2-transformed transition intensities using MSstats dataProcess function, with Tukey median polish (TMP) set as a summary method, followed by quantification (type = Sample).

### Secretome Analysis

The secretome of NSCs during both differentiations was analyzed by multiplex xMAP technology using the Cytokine 30-Plex Human Panel kit (Thermo Fisher Scientific, LHC6003M, full list of analytes in Supplementary Figure 1). The frozen conditioned medium was thawed on ice, 10 times concentrated using 3-kDa Amicon Ultra 2-mL centrifugal filters (Merck) at 4°C, and immediately analyzed to avoid repeated freeze-thaw cycles. An unconditioned basal NSC medium supplemented with EGF/FGF2 was used to dissolve and dilute calibration standards and as a background sample to an identical matrix between standards and samples. As this medium contained an external source of EGF and FGF2, data for these growth factors were excluded from further analysis. Each biological replicate was analyzed in 2 technical replicates. The assay was prepared according to the 30-plex kit manufacturer's instructions, and data were acquired on a Luminex 200 analyzer with xPonent software build 3.1.871.0 (Luminex Corp.) adequately calibrated according to the manufacturer's instructions. Fluorescence intensities of at least 100 beads per analyte were recorded. Raw median fluorescent intensity (MFI) values were exported from xPonent software, analyzed in R statistical environment (R Core Team, 2020), version 3.6. MFI values were normalized to cellular protein concentrations in corresponding culture dishes to minimize variability of cell counts in individual samples.

### Analysis of Differentially Expressed Proteins

Protein abundances from MSstats preprocessed SWATH-MS data were filtered to remove proteins with more than 28 missing values across all experimental conditions. In the remaining list of proteins, missing values were imputed before analysis as follows: for more than 50% missing values per condition, values were replaced by 1/3 of the lowest abundance of a given protein; for <50% missing values per condition, values were imputed using the k-nearest neighbor (knn) algorithm with *k* = 4. Linear models for microarray analysis (limma) R package (Smyth et al., 2020) were used to identify differentially expressed proteins. R package EGSEA (Alhamdoosh et al., 2017) was used to identify functional and pathway enrichment in the resulting list of differentially expressed proteins, according to the Gene Ontology (GO) vocabulary and Kyoto Encyclopedia of Genes and Genomes (KEGG) pathway list.

### Protein Co-expression Network Analysis

The protein co-expression network was constructed using weighted gene correlation network analysis as implemented in R package WGCNA (Langfelder and Horvath, 2008), using all quantifiable proteins from the SWATH-MS analysis as input data. We set the soft-thresholding power to 5 for adjacency matrix calculation. The protein expression dendrogram was cut into protein co-expression modules with 30 members set as the minimum module size. Module eigengenes (ME) were calculated as the principal component of each module, and modules were further simplified by merging based on the 0.25 cut height threshold (0.75 correlation of ME). To discover the possible relationships between protein expression profiles and observed phenotypes, we computed the correlation of ME with selected traits. *P*-values were derived by the Fisher transformation of each correlation. R package ComplexHeatmap (Gu et al., 2016) was used for data visualization, and package clusterProfiler (Yu et al., 2012) was used to identify module association with cell markers.

### Identification of Proteins With Similar Temporal Expression Pattern

The soft clustering method implemented in Mfuzz (Kumar and Futschik, 2007) R package was used to identify temporal protein expression patterns separately in spontaneously differentiated cells, and cells grown in BG differentiation conditions.

### RNA Isolation and cDNA Synthesis

Total RNA from cultured cells was harvested using RNeasy Plus Mini Kit (Qiagen) with cell homogenization with QIAshredder (Qiagen) according to the manufacturer's instructions. The concentration and quality of eluted RNA were determined with a NanoDrop spectrophotometer (Thermo Fisher Scientific). Two μg of RNA per sample were converted into cDNA with QuantiTect Reverse Transcription Kit (Qiagen) according to the manufacturer's instructions. Samples were then diluted to 25 ng/μL based on the previous concentration of RNA.

### Quantitative Real-Time PCR

Quantitative real-time PCR (qPCR) was used to analyze the relative gene expression of selected markers. The volume of each reaction was 20 μL containing 4 μL of 5 × HOT FIREPol EvaGreen qPCR Mix Plus (Solis BioDyne), 125 nM of each primer (Supplementary Table 1A), and 25 ng of cDNA template and PCR water. The CFX96 Touch Real-Time detection system (Bio-Rad) was used to monitor amplification with the following settings: 12 min at 95°C for enzyme activation, then 15 s at 95°C for template denaturation followed by 40 cycles of 30 s at 57°C for primer annealing and 30 s at 72°C for an extension. Endogenous control (housekeeping markers), glyceraldehyde-3-phosphate dehydrogenase (GAPDH), and ATP synthase subunit beta, mitochondrial (ATP5F1B), were used for normalization of cycle threshold (Ct) values.

### Immunocytochemistry

NSCs were cultivated on LabTek II CC^2^ (Thermo Fisher Scientific) coated with 20 μg/mL poly-L-ornithine and 5 μg/mL laminin and differentiated as described above. Cells were collected at day 0 (proliferating NSCs) and at days 7, 14, 21, and 28 from both S and BG differentiation protocols.

Cells were briefly rinsed with PBS, fixed with 4% paraformaldehyde in PBS for 10 min, and permeabilized with 0.1% Triton X-100 in PBS for 10 min. Cells were incubated for 10 min in 50 mM NH_4_Cl in PBS to remove the free aldehyde groups, blocked in 5% goat serum in PBS for 1 h, and incubated with primary antibodies (rabbit monoclonal anti-SOX2, Cell Signaling, 3579S, 1:400; mouse monoclonal anti-Nestin, Cell Signaling, 33475S, 1:800; rabbit monoclonal anti-S100B Abcam, ab52642, 1:400; mouse monoclonal anti-TUBB3, Exbio, 11-264-C100, 1:500) diluted in PBS/0.01% BSA for 3 h. After incubation with primary antibodies, cells were washed 3 times in PBS/0.05% Tween. Secondary antibodies labeled with AlexaFluor488 (goat anti-mouse IgG, A28175, 1:500) and AlexaFluor647 (goat anti-rabbit IgG, A32733, 1:1,000) were mixed with DAPI (1 μg/mL) to mark nuclei. Cells were incubated in secondary antibodies for 45 min in the dark. Subsequently, cells were washed 3 times in PBS/0.05% Tween and 5 min in PBS. All incubations were performed at room temperature. Cells were coverslipped in ProLong Glass Antifade Mountant (Thermo Fisher Scientific, P36980). Images were acquired on a Leica TCS SP5 confocal microscope and processed and analyzed in Fiji.

### Immunoblotting

Cells were lysed in ice-cold RIPA buffer (150 mM NaCl; 5 mM EDTA, pH 8; 50 mM Tris–HCl, pH 7.4; 0.5% NP-40; 1% sodium deoxycholate; 1% Triton X-100 and 0.1% SDS) with 1× Halt protease and phosphatase inhibitor cocktail (Thermo Fisher Scientific). Lysates were sonicated in an ice bath, and unlysed debris was pelleted by centrifugation for 10 min at 16,000 g at 4°C. The protein concentration in supernatants was determined by BCA assay (Thermo Fisher Scientific). Recombinant human VEGF121 and VEGF165 proteins (same as in the case of VEGF induction; 1 ng) were used as positive controls for the VEGF121 antibody.

Three μg of cellular proteins (6 μg for VEGF and HIF1-α detection) were separated in 4–12% Bis–Tris or 3–8% Tris-acetate gradient NuPAGE gels (Invitrogen) under reducing conditions according to the manufacturer's instructions. Proteins were transferred by the Trans-Blot Turbo transfer system (Bio-Rad) to the nitrocellulose (or PVDF for VEGF121 antibody) membranes. Membranes were blocked for 1 h at room temperature and incubated overnight at 4°C with a primary antibody (see Supplementary Table 1B for details). Membranes were washed 3 times for 10 min in Tris-buffered saline with 0.05% Tween 20 (TTBS) and incubated for 60 min at room temperature with the appropriate secondary antibody diluted 1:10,000 in 5% dry skim milk in TTBS. Membranes were washed in TTBS and incubated in ECL Prime reagent (Amersham). The Chemidoc XRS+ detection system with Image Lab (version 5.2.1 build 11) software (Bio-Rad) was used to detect the chemiluminescent signal.

### Statistical Analysis

All statistical analyses were performed in the R statistical environment (R Core Team, 2020). Statistical analysis of proteomic data is described separately in the previous paragraphs. For qPCR data, Luminex secretome analysis and influence of VEGF stimulation on growth and survival of NSCs in culture, linear mixed models with differentiation protocol vs. time in culture as factors were fitted to the data; marginal means and confidence intervals were estimated using emmeans R package (Lenth, 2020); and multiple-testing adjusted *p*-values for individual pairwise comparisons were derived using the Tukey HSD test.

## Results

### Neural Stem Cell Differentiation

**Figure 1:**
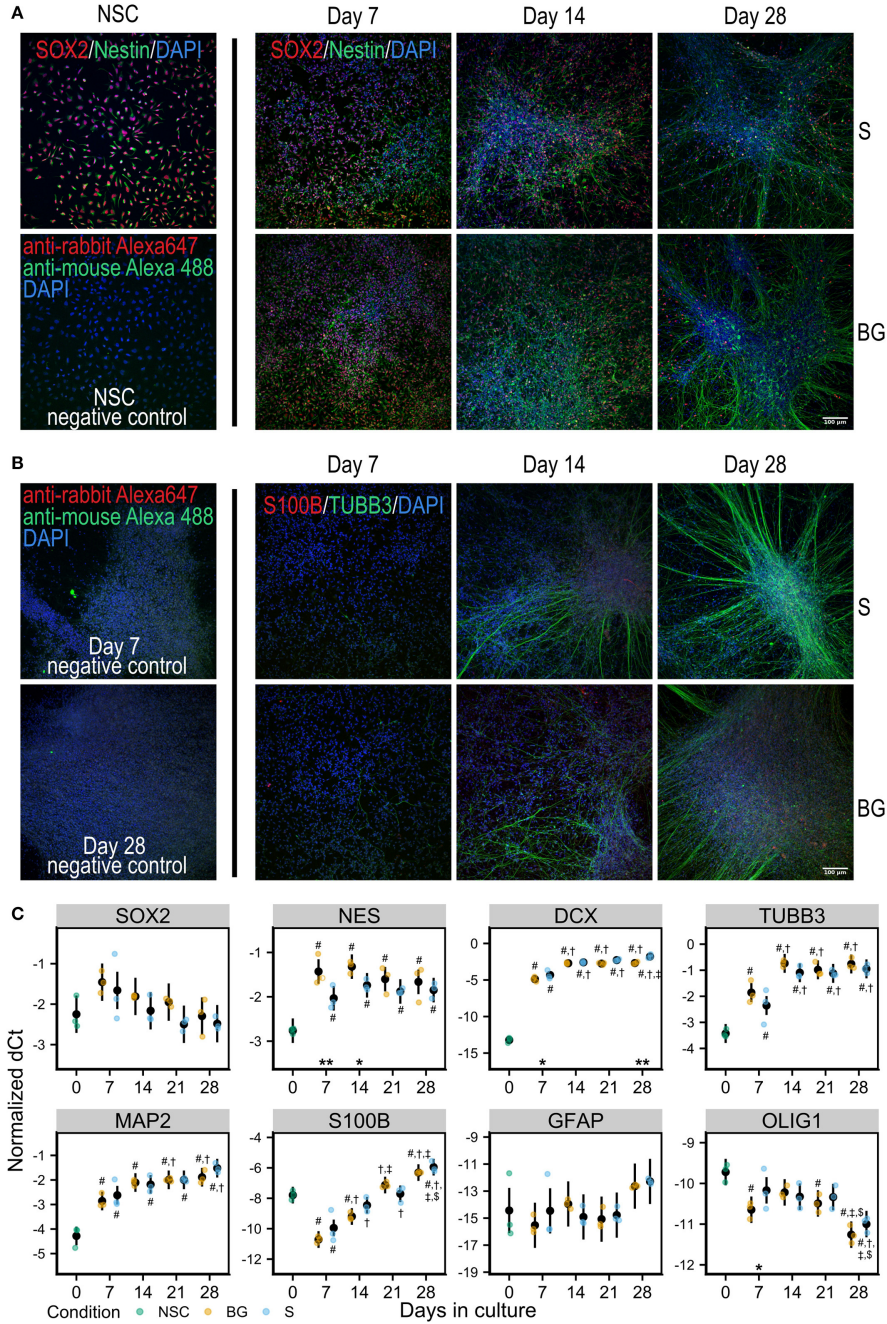
Differentiation of NSCs *in vitro*. **(A)** Immunofluorescence staining for NSC markers Nestin (green) and SOX2 (red) in NSCs and during S or BG differentiation. **(B)** Immunofluorescence staining for neural marker TUBB3 (green) and glial marker S100B (red) during S or BG differentiation. Cell nuclei are counterstained by DAPI (blue) in both panels. Negative controls (omitted primary antibodies) for NSCs are shown in **(A)** and for days 7 and 28 of S differentiation in **(B)**. **(C)** Expression of transcripts of selected cell type markers during S and BG differentiation by RT-qPCR. dCt values for individual transcripts normalized to the average of two housekeeping controls (GAPDH and ATP5F1B) from three independent experiments are shown as individual points, estimated model mean ± 95% confidence intervals shown as black points and lines. Statistical significance was calculated from the linear mixed model of differentiation protocol (BG vs. S) and day in culture (0, 7, 14, 21, 28) within each experiment, with Tukey adjusted *p*-values for all pairwise comparisons. Asterisks above the x axis denote significant difference between BG and S protocol at given day (^*^p < 0.05, ^**^p < 0.01). Symbols above (BG) and below (S) data points denote significant difference (*p* < 0.05) of given group vs. day 0 (#), or day 7 (†), day 14 (‡), and day 21 ($) of the same differentiation protocol.

Human H9 NSCs were differentiated spontaneously by the withdrawal of EGF and FGF2 from the cell culture media (S differentiation) or spontaneously with trophic support by a combination of neurotrophic factors BNDF and GDNF (BG differentiation) for 28 days *in vitro* (Figures 1A,B).

At the level of mRNA, both types of differentiation showed similar trends of expression of selected markers of NSCs or differentiated neural cells (Figure 1C). NSC marker transcription factor SOX-2 (SOX2) was not significantly changed during differentiation, and NSC marker Nestin (NES) was increased during differentiation, compared to day 0, showing a trend for decrease from day 7 to day 28. The expression of neuronal markers neuronal migration protein doublecortin (DCX), tubulin β-3 chain (TUBB3), and microtubule-associated protein 2 (MAP2) was strongly increased at day 7, compared to day 0, and was rising during the whole differentiation. Glial marker protein S100-B (S100B) sharply decreased at day 7 compared to day 0 and then continuously increased with time in culture. Astrocyte-specific marker glial fibrillary acidic protein (GFAP) was detected at very low levels and was not significantly changing. The marker of oligodendrocytes oligodendrocyte transcription factor 1 (OLIG1) was decreasing through the differentiation.

### Proteome Analysis

**Figure 2:**
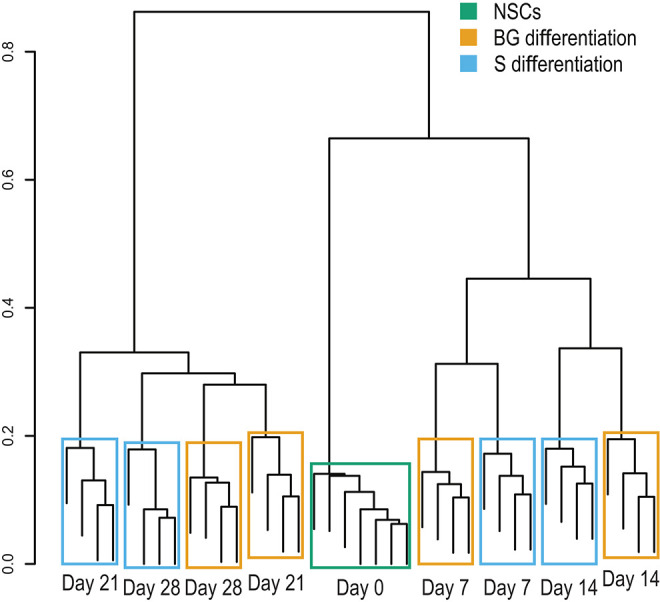
Sample clustering based on protein abundances from SWATH-MS analysis. Hierarchical clustering of individual biological replicates based on SWATH-MS-quantified proteins shows samples primarily co-clustering based on time in culture (NSC, day 7, day 14, and so on) and secondarily based on employed differentiation protocol.

The cellular proteome during S and BG differentiation at days 0, 7, 14, 21, and 28 was analyzed using the SWATH-MS method. The sample-specific library resulted in the identification of 23,645 unique peptides assigned to 2,870 human proteins with minimal two peptides and three transitions per peptide. In total, abundances of 2,804 proteins were obtained by SWATH-MS data summarization in MSstats (Supplementary Table 1C). We performed cluster analysis of SWATH-MS data based on abundances of quantified proteins, which revealed the co-clustering of samples from S and BG differentiation at days 7 and 14 and divergence between both differentiations at days 21 and 28 (Figure 2). These results show that differences between samples in the early stages of NSC differentiation are caused mostly by the differentiation time course rather than the type of differentiation itself. However, differentiation type influences the later stages of NSC differentiation.

*In vitro* NSC differentiation was accompanied by large-scale changes in the global cellular proteome (1,359 and 1,446 proteins changed between day 0 and day 28 of S and BG differentiation, respectively) (Table 1). Major changes in protein expression occurred between day 0 and day 7 of both differentiations-−1,024 and 1,057 changed proteins in case of S and BG differentiation, respectively. In contrast, 242 and 254 proteins were differentially expressed between day 14 and day 28 of S or BG differentiation, respectively. Numbers of differentially expressed proteins between both types of differentiation were relatively low (e.g., 107 proteins at day 28).

**Table 1:** Numbers of differentially expressed proteins between differentiation time-points from total number of 2,515 proteins subjected to analysis.

	**Day 0**				**Day 7**	**Day 14**	**Day 7**	**Day 14**	**Day 21**	**Day 28**
**Differentiation**	**Day 7**	**Day 14**	**Day 21**	**Day 28**	**Day 21**	**Day 28**	**Day 7**	**Day 14**	**Day 21**	**Day 28**
S	1,024	1,145	1,279	1,359	450	242	71	87	125	107
BG	1,057	1,197	1,336	1,446	546	254				

*Only proteins with fold change ≥ 2 and multiple-testing adjusted p ≤ 0.05 as analyzed in limma R package were counted. The list of all differentially expressed proteins across the conditions examined is provided as Supplementary Table 1D*.

### Secretome Analysis

**Figure 3:**
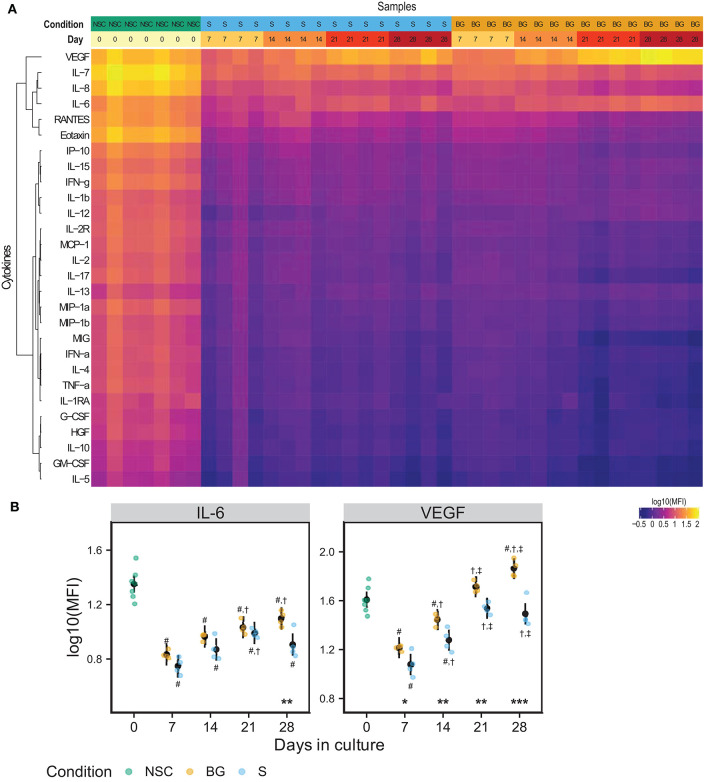
NSC secretome. **(A)** Heatmap of log10(MFI) values normalized to the protein content of secreting cells. **(B)** Graphs of the normalized log10(MFI) values (points) and estimated model mean ± 95% confidence intervals (black points and lines) for IL-6 and VEGF during NSC differentiation. Statistical significance was calculated from a linear mixed model of differentiation protocol (BG vs. S) and days in culture (0, 7, 14, 21, 28) within each experiment, with Tukey adjusted *p*-values for all pairwise comparisons. Asterisks above the x axis denote significant difference between BG and S protocol at a given day (^*^p < 0.05, ^**^p < 0.01, ^***^p < 0.001). Symbols above (BG) and below (S) data points denote significant difference (*p* < 0.05) of given group vs. day 0 (#), or day 7 (†), and day 14 (‡) of the same differentiation protocol.

We analyzed levels of 28 secreted growth factors, chemokines, and cytokines in a medium conditioned for 48 hours in the presence of NSCs during the differentiation time course. Figure 3A shows a heatmap of median fluorescence intensities (MFI) from xMAP analysis of the conditioned medium. IL-6 and VEGF concentrations were consistently growing with time in culture, in both differentiation protocols used (Figures 3A,B). At day 28, both IL-6 and VEGF levels were significantly higher in the medium from cells differentiated by BG protocol compared to S protocol. The other analytes were detected at the background levels. Due to normalization to cellular protein concentrations (~6 times higher in differentiating cell samples), the MFI values of analytes secreted by proliferating NSCs (day 0) may be overestimated. The complete MFI data for all analyzed cytokines are provided in Supplementary Table 1E and graphs of log10 (MFI) values in Supplementary Figure 1.

### Protein Co-expression Network Analysis

**Figure 4:**
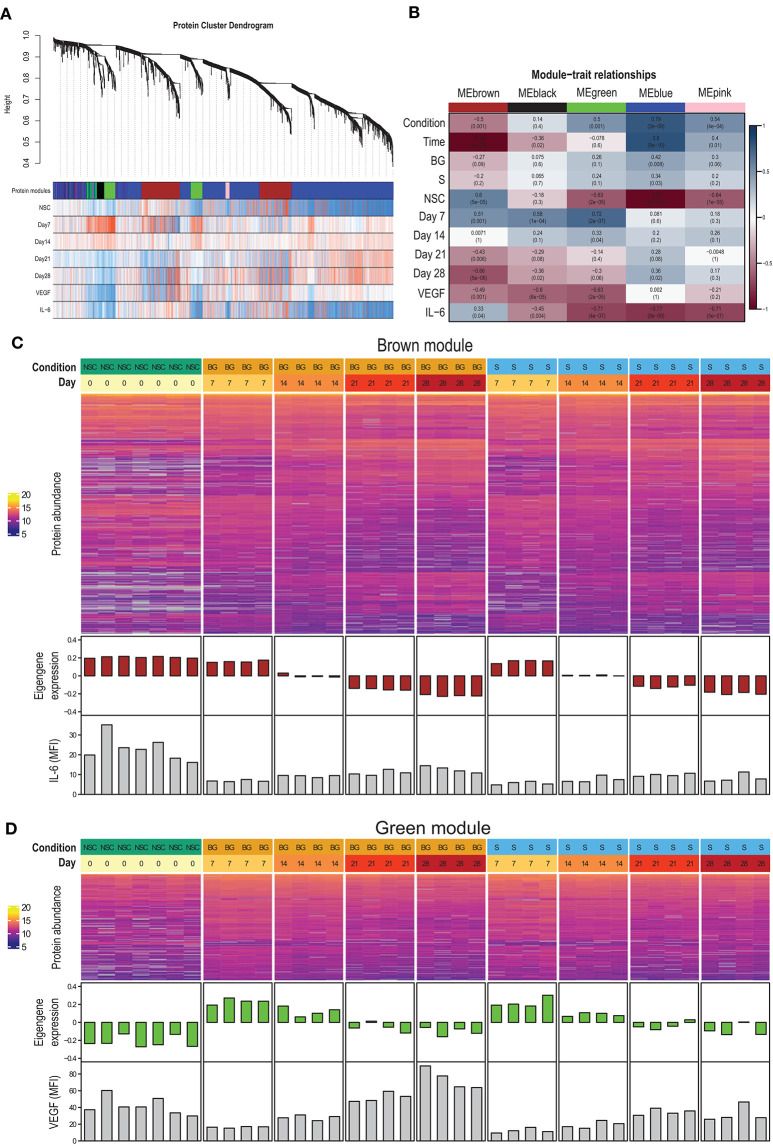
Protein co-expression network. **(A)** The dendrogram shows the hierarchical clustering of proteins into co-expression modules (shown as color-coded blocks). The heatmaps below show the correlation of protein expression with the particular phenotypic trait (red—positive correlation, blue—negative correlation). **(B)** Correlation of module eigengenes with phenotypic traits. **(C)** Heatmap of brown module protein abundance, together with module eigengene expression in individual samples and secreted IL-6 levels. **(D)** Heatmap of green module protein abundance, together with module eigengene expression in individual samples and secreted VEGF levels.

To identify proteins with similar expression patterns and relate these patterns to observed phenotypes, we constructed a protein co-expression network using the WGCNA method. The largest of the identified co-expression modules (blue) consisted of proteins with low expression at the NSC stage and increasing over the time course of differentiation (Figures 4A,B; Supplementary Figure 2). The brown module contained proteins expressed at the NSC stage and decreasing over time (Figures 4A–C). This module also correlated with IL-6 secretion. The green module contained proteins with low expression at the NSC stage, increasing at an early stage of *in vitro* differentiation (day 7–day 14) and declining expression at later stages (day 21–day 28) (Figures 4A,B,D). This module also had a strong negative correlation with VEGF secretion, as this was at its lowest at day 7 (Figures 3B, 4D). Complete data for protein membership in identified co-expression modules and their correlation with traits is provided as Supplementary Table 1F. Next, we used R package clusterProfiler to analyze whether proteins positively correlated with particular phenotypic traits within a given module correspond to known markers of selected cell types and lineages. We found that proteins with expression upregulated at day 7 within green and brown modules and downregulated at day 28 within the brown module best correspond to the “Embryonic pre-frontal cortex, Normal, Neural progenitor cell” type, while proteins upregulated at day 28 within blue and brown modules best correspond to the “Embryonic prefrontal cortex, Normal, Astrocyte” cell type. This finding is surprising, given that only limited signs of differentiation into glial cells were observed in H9 NSCs in our other experiments.

### Identification of Proteins With a Similar Temporal Expression Pattern

Next, we used soft clustering of proteins into groups with similar temporal expression profiles implemented in Mfuzz R package to further explore regulation of protein expression in time. By clustering separately proteins quantified in S or BG differentiation, we obtained 28 clusters for each condition (Supplementary Table 1G, Supplementary Figure 3).

**Figure 5:**
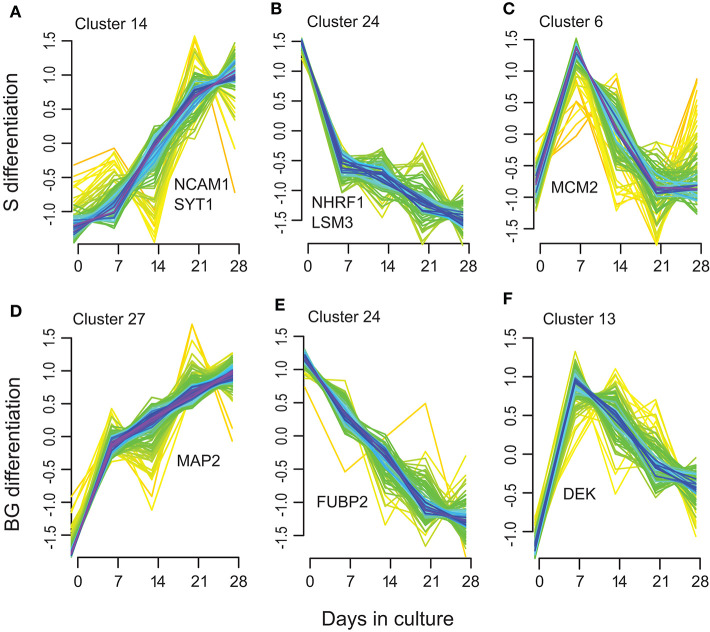
Selected examples of the Mfuzz clustering results. **(A,D)** representing Mfuzz clusters showing an increase in protein abundance over the S **(A)** and BG **(D)** differentiation with representative proteins for these clusters: NCAM1, SYT1, and MAP2. **(B,E)** representing Mfuzz clusters showing a decrease in protein abundance over the S **(B)** and BG **(E)** differentiation with representative proteins for these clusters: NHRF1, LSM3, and FUBP2. **(C,F)** as examples of clusters with maximum protein abundance at day 7 of S **(C)** or BG **(F)** differentiation with representative proteins for these clusters: MCM2 and DEK.

Among these clusters, clusters 7, 14, 15, 17, 20, and 23 showed an increase in protein abundance over time in the S differentiation and clusters 2, 5, 7, 12, 14, and 27 in BG differentiation. In total, 126 proteins were assigned to these BG differentiation clusters, respectively, 100 proteins to S differentiation clusters. Among them are represented proteins involved in brain/nervous system development including synaptotagmin-1 (Figure 5A), neuronal membrane glycoprotein M6-a, serine/threonine-protein kinase DCLK1, dihydropyrimidinase-related protein 2, MAP2 (BG differentiation only) (Figure 5D), or DCX (S differentiation only); axon guidance/axonogenesis proteins like neural cell adhesion molecule 1 or microtubule-associated protein 6; proteins involved in apoptosis-like macrophage migration inhibitory factor, cathepsin D, or caspase-3 (BG differentiation only); transport proteins like sideroflexin-3, alpha-centractin, cytoplasmic dynein 1 heavy chain 1, or endoplasmic reticulum-Golgi intermediate compartment protein 1; and proteins involved in Wnt signaling like catenin β-1 (S differentiation only) or protein wnt less homolog (BG differentiation only).

Another group of clusters, 9, 18, 19, 22, and 24 in S differentiation (60 proteins) and clusters 8, 18, 20, 21, and 24 in BG differentiation (69 proteins) showed a decrease in protein abundance over time. Proteins decreasing over the differentiation time course included proteins involved in mRNA processing/splicing, including far upstream element-binding protein 2 (Figure 5E) or U6 snRNA-associated Sm-like protein LSm3, and regulation of translation like SAP domain-containing ribonucleoprotein and apoptotic signaling (Wnt signaling) like Na(+)/H(+) exchange regulatory cofactor NHE-RF1 (Figure 5B).

Clusters with maximum protein abundance at day 7; clusters 2, 6, 8, 11, and 27 in S differentiation (72 proteins); and clusters 11, 13, 17, 23, and 26 (44 proteins) in BG differentiation overlap with the green module in WGCNA analysis (32 proteins in S differentiation and 21 proteins in BG differentiation). These proteins are again mainly involved in regulation of mRNA binding or cell cycle and interestingly DNA repair (e.g., DNA replication licensing factor MCM2, protein DEK, DNA ligase 3) (Figures 5C,F).

### Functional Annotation of Differentially Expressed Proteins Using KEGG and GO

Lists of proteins quantified by SWATH-MS and detected as significantly differentially expressed between particular time points or differentiation protocols were subjected to functional analysis using Kyoto Encyclopedia of Genes and Genomes (KEGG) pathways and Gene Ontology (GO) biological processes.

**Figure 6:**
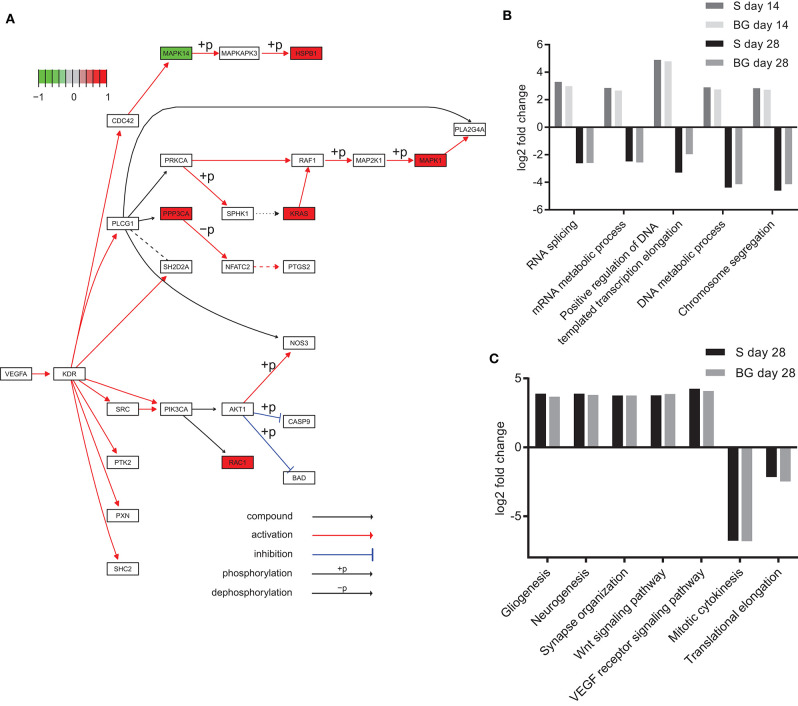
Functional analysis of SWATH-MS data. **(A)** KEGG diagram of VEGF pathway regulation and changes in abundance of pathway members identified in our experiments. Heat shock protein beta-1 (HSPB1), serine/threonine-protein phosphatase 2B catalytic subunit alpha isoform (PPP3CA), GTPase KRas (KRAS), mitogen-activated protein kinase 1 (MAPK1), and Ras-related C3 botulinum toxin substrate 1 (RAC1) were upregulated, whereas mitogen-activated protein kinase 14 (MAPK14) was downregulated at day 28 of BG differentiation in comparison to day 0. **(B)** Selected biological processes (GO) upregulated at day 14 and downregulated at day 28 of both differentiations. Log2 fold change comparison between day 0 and day 14 or 28 of S and BG differentiation with fold change ≥ 2 and *p* ≤ 0.05. **(C)** Upregulation and downregulation of selected terms in biological processes of the GO section in differentiated cells. Log2 fold change comparison between day 0 and day 28 of S and BG differentiation for selected GO biological processes with fold change ≥ 2 and *p* ≤ 0.05. Differentially expressed proteins were identified with Limma R package, and KEGG/GO annotations were mapped with R package EGSEA.

Analysis of KEGG pathways revealed downregulation of spliceosome and ribosome biogenesis in eukaryotes at day 28 of both differentiations, compared to day 0. Interestingly, these processes were upregulated at day 14, compared to both days 0 and 28. We also observed the downregulation of DNA replication in the case of BG differentiation, compared to both days 0 and 14, whereas it was upregulated in S differentiation at day 28, compared to day 0. On the other hand, global metabolism (e.g., glycolysis or fatty acid metabolism pathways), RNA degradation, regulation of actin cytoskeleton, axon guidance, protein processing in endoplasmic reticulum, protein export, synaptic vesicle cycle, and HIF-1 signaling pathway were upregulated at both days 14 and 28, and the VEGF signaling pathway was upregulated at day 28 of both differentiations, compared to day 0 (Figure 6A). A complete list of identified KEGG pathways is in Supplementary Table 1H.

Our GO analysis of biological processes supported results from the KEGG pathways and Mfuzz clustering analysis. Similarly to KEGG results, processes of RNA splicing, mRNA metabolic process, chromosome segregation, and DNA metabolic process were upregulated at the day 14 but downregulated at day 28, compared to day 0 (Figure 6B). Mitotic cytokinesis was downregulated at both days 14 and 28, compared to day 0. In contrast, at days 14 and 28, lipid metabolic process, regulation of cytoskeleton organization, response to oxidative stress, regulation of synaptic vesicle transport, Wnt signaling pathway, VEGF receptor signaling pathway, regulation of neuron death, gliogenesis, and neurogenesis were upregulated (Figure 6C). A complete list of identified GO biological processes is in Supplementary Table 1I.

### Validation of SWATH-MS Protein Quantification by Immunoblotting for Selected Markers of NSCs and of Neural Differentiation

**Figure 7:**
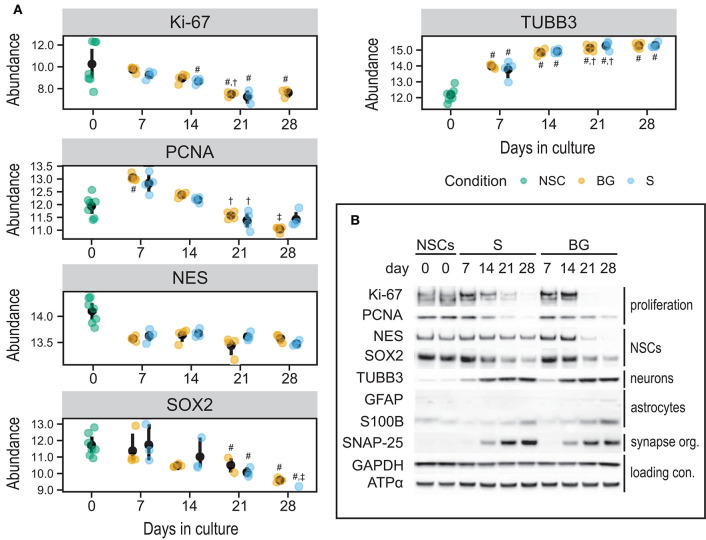
Expression of selected cell type markers during S and BG differentiation. **(A)** SWATH-MS results of protein abundances of Ki-67, PCNA, NES, and SOX2 showed a decrease of proliferation and NSC markers, whereas NPC/neuronal marker TUBB3 was increased. Symbols denote significant difference (*p* < 0.05, moderated t-statistic from limma) of a given group vs. day 0 (#), or day 7 (†), and day 14 (‡) of the same differentiation protocol. **(B)** Immunodetection of selected NSC, NPC, neuronal, and glial markers. Markers of proliferating cells (Ki-67, PCNA) and undifferentiated NSCs (NES, SOX2) were expressed predominantly in proliferating cells (NSCs, day 0) and decreased during both differentiations. On the other hand, markers of differentiating neurons (TUBB3) and astrocytes (GFAP, S100B) increased during both differentiations, but the expression of astrocyte markers was overall very low. GAPDH and ATPase subunit alpha (ATPα) were used as loading controls. Representative images from two biological replicates are shown.

To validate quantitative SWATH-MS data (Figure 7A) and to confirm the differentiation state of cells during S and BG differentiation, levels of selected proteins were analyzed by immunoblotting. Specifically, markers of NSCs (NES and SOX2), neuronal progenitor cells (NPCs)/neurons (TUBB3), or glial cells (S100B and GFAP), and markers of cellular proliferation proteins Ki-67 and proliferating cell nuclear antigen (PCNA) were monitored during the time course of both differentiation protocols at days 0, 7, 14, 21, and 28. Abundances of markers of proliferating cells (Ki-67 and PCNA) as well as markers of NSCs (NES and SOX2) were decreasing over time in both S and BG differentiations (Figure 7B), similarly to SWATH-MS data. On the other hand, the expression of TUBB3, a marker of NPCs and neurons, was increasing from day 7. Ongoing neuronal differentiation was further supported by increased expression of synaptosomal-associated protein 25 (SNAP-25) from day 14 of both differentiations (Figure 7B), which is in agreement with upregulation of synapse organization identified by GO analyses (Figure 6C) at day 28 of both differentiations. Typical markers of glial cell lineages, including GFAP, S100B, and OLIG1, were not among proteins quantified by SWATH-MS, confirming our observation from immunofluorescence (Figure 1B; no signal was detected for GFAP and OLIG1, data not shown) and RT-qPCR analyses (Figure 1C) of limited differentiation potential of H9 NSCs into glial cells. For this reason, we aimed to confirm or rule out the expression of GFAP and S100B by immunoblotting. While levels of S100B were detectable and growing over time, expression of a marker of astrocytes, GFAP, was detectable only in BG differentiation from day 14 (Figure 7B, Supplementary Figure 4). Based on these results, both differentiations led to differentiation into NPCs or neurons, while some cells were still expressing NSC markers.

### Role of the VEGF Pathway in Cellular Proliferation, Differentiation, and Survival of NSCs During *in vitro* Culture

**Figure 8:**
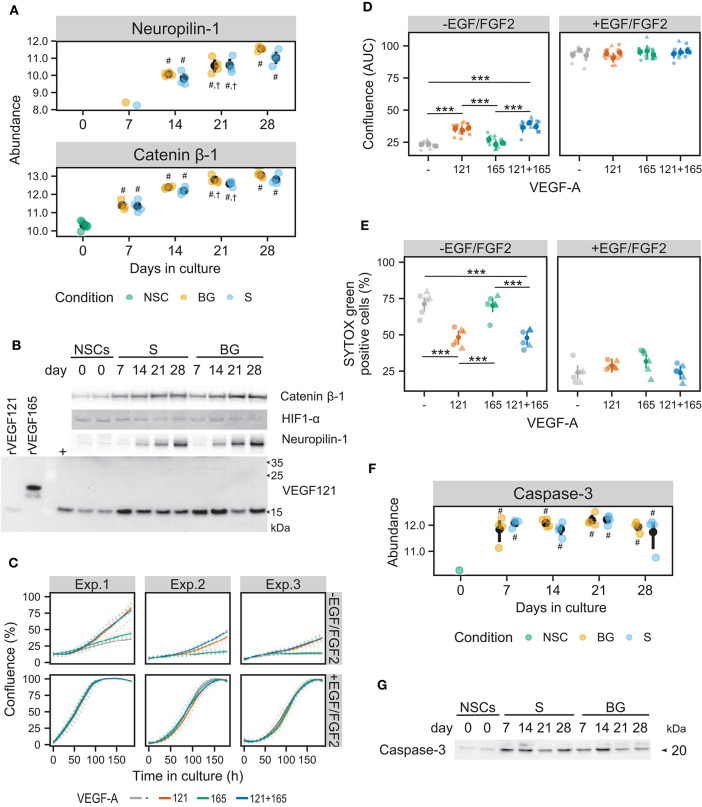
Influence of the VEGF pathway on growth and survival of NSCs in culture. **(A)** SWATH-MS results show an increase of protein abundances of neuropilin-1 and catenin β-1 during S and BG differentiation. Symbols denote significant difference (*p* < 0.05, moderated t-statistic from limma) of a given group vs. day 0 (#), or day 7 (†) of the same differentiation protocol. **(B)** Expression of catenin β-1, HIF1-α, neuropilin-1, and VEGF121 during both differentiations detected by immunoblotting. rVEGF121 and rVEGF165—recombinant proteins. Symbol + denotes mix of proliferating and differentiated NSCs used as a positive control. **(C)** Cell growth is depicted as a change in confluence (acquired and analyzed on an IncuCyte incubator microscope) in time. Data from 3 independent experiments with 6 wells in each experimental condition are shown. Full lines indicate a smooth average over 6 wells for a given condition, dotted lines data from individual wells. **(D)** The area under the growth curve was calculated independently for each well and is displayed as mean (dot) and confidence interval (line) for each of the three experiments. Statistical significance was calculated by fitting a linear mixed model of VEGF and EGF/FGF2 supplementation within each experiment and is denoted with ^***^ (Tukey adjusted *p* < 0.001) in the given pairwise comparison. **(E)** Cell damage, as indicated by SYTOX Green positivity. After 8 days of cultivation, cells were treated with vital and dead cell staining and captured images were analyzed in Fiji. Data from two independent experiments with 3 wells per condition and 3 images per well are shown. Statistical significance was calculated from a linear mixed model of VEGF and EGF/FGF2 supplementation within each experiment, with Tukey-adjusted p-values for pairwise comparison and denoted with ^***^ (*p* < 0.001). **(F)** SWATH-MS results show an increase of protein abundance of caspase-3 at day 7 in both differentiation protocols. Symbols denote significant difference (*p* < 0.05, moderated t-statistic from limma) of a given group vs. day 0 (#). **(G)** Detection of activated caspase-3 during S and BG differentiation confirmed its increase at days 7 and 14 in comparison to day 0. Immunoblots shown in **(B,G)** are representative images from two biological replicates. Corresponding loading controls (GAPDH and ATPα) are shown in Figure 7B.

As the secretome analysis showed an increase in VEGF secretion with ongoing cell differentiation, we decided to focus on a deeper analysis of the role of the VEGF pathway in the survival, proliferation, and differentiation of H9 NSCs. The VEGF antibody used in the xMAP multiplex assay detects VEGF-A isoforms 121 (VEGF121) and 165 (VEGF165), but binding to other VEGF proteins was not verified by the manufacturer. Thus, we attempted to analyze VEGF-A production by NSCs and differentiating cells also by immunoblotting, which should allow distinguishing isoforms based on their molecular weight. However, post-translational modifications (N-glycosylation and dimerization via disulfide bonds) together with many possible isoforms of VEGF-A (Woolard et al., 2009) may affect proper identification of VEGF121 and VEGF165. Therefore, we employed recombinant proteins VEGF121 and VEGF165, which were detected at expected molecular weights, showing that the VEGF121 antibody is not specific for VEGF121 only. VEGF121 protein (likely N-glycosylated according to a slight mass shift in comparison to recombinant VEGF121) was detected in all samples with the highest expression at days 7 and 14 (Figure 8B). Surprisingly, VEGF121 expression in cells was decreased at days 21 and 28, even though it was still higher than at day 0, suggesting that either cellular and secreted VEGF121 levels do not directly correspond, or other VEGF-A isoforms were quantified together during secretome analysis. Moreover, we did not detect any expression of VEGF165 at any time point of both types of differentiation.

Increased expression of neuropilin-1, known as a receptor for VEGF-A in neurons (Tillo et al., 2015), was observed from day 14 in SWATH-MS data as well as on immunoblot in both types of differentiation (Figures 8A,B).

Increased abundance of catenin β-1 from day 7 of both differentiations (Figure 8B) confirmed results from SWATH-MS quantification (Figure 8A) and is in agreement with the upregulation of the Wnt signaling pathway (Figure 6C), which may also be involved in VEGF expression regulation. Activation of the HIF-1 signaling pathway was supported by the presence of hypoxia-inducible factor 1-α (HIF1-α) at both differentiations. However, abundance of HIF1-α was decreased at days 21 and 28 of both differentiations, suggesting its role mainly in early stages of NSC differentiation (Figure 8B).

To analyze the effect of VEGF-A isoforms 121 and 165 on neural cells directly, we cultivated NSCs in the proliferation medium with EGF and FGF2 or in the medium for S differentiation with supplementation of VEGF121 and/or VEGF165. Cell proliferation potential was analyzed by monitoring of cell confluency using time-lapse live cell microscopy for 8 days *in vitro*. At the experiment end point, cell viability was analyzed by staining for damaged and dead cells.

Cell confluency analysis revealed that in a condition of S differentiation, VEGF121 itself or in combination with VEGF165 significantly induced cellular proliferation (~1.5-fold change based on area under the growth curve), compared to control and VEGF165 (Figures 8C,D). VEGF165 supplementation alone had no significant effect (Figures 8C,D). On the contrary, we did not observe any beneficial effect of VEGF121 and/or VEGF165 on NSC proliferation in the presence of EGF/FGF2.

To assess the effect of VEGF121 and VEGF165 on cell viability, we stained cells with SYTOX Green dye, which enters the cells with compromised plasma membrane integrity and labels cell nuclei. To obtain total cell counts, vital dye Hoechst 33342 was used to counterstain all nuclei. VEGF121 itself or in combination with VEGF165 significantly suppressed cell death/damage in the condition of S differentiation (48.31 ± 10.23% and 47.52 ± 12.23%, respectively, compared to 71.83 ± 10.66% SYTOX Green-positive cells in control) (Figure 8E). In contrast, VEGF165 itself did not affect cellular viability (70.14 ± 9.91% SYTOX Green-positive cells). We did not find any benefit of VEGF121 and/or VEGF165 to cell survival in the case of NSC cultivation in the medium with EGF/FGF2. These results show that VEGF121 can induce cellular proliferation and reduce cell damage or death of human NSCs in the absence of other growth factors, whereas VEGF165 has no such effect, at least in *in vitro* conditions.

Increased abundance of activated caspase-3 (marker of apoptosis) was identified in SWATH-MS data (Figure 8F) and validated by immunoblotting in both differentiation protocols from day 7 but more pronounced in S differentiation (Figure 8G). This result suggests that trophic support (BDNF and GDNF) is important for cellular viability after the change of environment, i.e., withdrawal of growth factors from the proliferation medium and VEGF secretion by differentiating cells might act as an additional pro-survival stimulus, counteracting activation of programmed cell death.

## Discussion

In the present study, we differentiated human H9 NSCs by withdrawal of growth factors (EGF/FGF2) to simulate dramatic change of the microenvironment after cell grafting or with neurotrophic support of BDNF/GDNF, which were previously shown to support survival of transplanted cells (Wang et al., 2011; Rosenblum et al., 2015). Immunocytochemistry and gene expression analyses revealed that both S and BG differentiations led mainly to cells of neuronal lineage, with only very limited differentiation into cells expressing glial markers, which was further confirmed by immunoblotting. These results are in agreement with data from Bohaciakova et al. who observed a very low expression of GFAP in H9 ESC-derived NSC differentiated to astrocytes for up to 6 weeks (Bohaciakova et al., 2019). We did not identify significant changes in expression of selected neuronal markers between S and BG differentiation.

We then focused on temporal changes in the cellular proteome during both differentiations. SWATH-MS analysis proved that global changes in the cellular proteome occur at the very early stage of both differentiation protocols likely as a response to a dramatic change of the microenvironment. Interestingly, hierarchical clustering of samples based on protein abundances suggested divergence in protein expression profiles at the later stages of S and BG differentiation. The number of significantly differentially expressed proteins at the same time point between the two protocols was low but mildly growing over time.

Proteomic and transcriptomic data are not fully comparable, because the expression of a specific protein may be regulated differentially at the mRNA and protein levels. A study of gene expression control in mouse fibroblasts revealed that abundances of cellular proteins are mostly regulated at the translational level, as the average protein has about five times longer half-life than the average mRNA and its translation rate is in general ~70 times higher than the transcription rate (Schwanhäusser et al., 2011). This biological phenomenon was further confirmed in studies comparing the transcriptome and proteome of differentiating human ESCs during the formation of embryoid bodies (Fathi et al., 2009) and during neural differentiation of human ESCs (Fathi et al., 2014). In our experiments, we noted a discrepancy in expression of NSC markers NES and SOX2 as assayed by RT-qPCR vs. protein-based methods. According to our results from immunocytochemistry, SWATH-MS, and immunoblotting, both NES and SOX2 were clearly detectable at high levels in NSCs, and although their expression had a decreasing trend, it persisted at all stages of both S and BG differentiations. Whereas, results of RT-qPCR, SWATH-MS, and immunoblotting reflect an expression in the whole population, immunocytochemistry revealed that some strongly NES- or SOX2-positive cells remain in culture till day 28, but there are also many cells completely negative for these proteins. On the contrary, most NSCs have a uniform NES- or SOX2-positive staining pattern. Although NES is broadly used as a marker of NSCs, it also affects the proliferation and cell death of NPCs or axonogenesis in immature neurons [reviewed in (Bott and Winckler, 2020)]. Therefore, its presence during the whole differentiation is biologically relevant. The discrepancies between our transcriptomic and proteomic data may be further accented by different kinds of data normalization for each method. Global normalization was used for SWATH-MS data, and we observed that majority of quantified proteins changed its abundance during differentiation, mainly between days 0 and 7, including proteins of metabolic pathways. Zheng et al. previously reported transition from glycolysis in NPCs to oxidative phosphorylation in differentiating cells during neuronal differentiation of human NPCs (Zheng et al., 2016). This makes selection of housekeeping genes for RT-qPCR experiments challenging, as highly expressed cytoskeletal or metabolic genes are usually employed and both of these classes can change during cell differentiation. In the end, we selected two housekeeping genes, GAPDH (enzyme of glycolysis) and ATP5F1B (subunit of ATP synthase—oxidative phosphorylation), as our RT-qPCR normalizers. Based on observed large differences in the cell proteome profile between days 0 and 7 together with the abovementioned published switch in metabolic pathways, it is possible that normalization based only on two selected genes influences the apparent expression of NES and SOX2, especially when comparing day 0 (NSC stage) to later stages with differentiating cells. On the other hand, for comparisons among day 7–day 28, we see an overall good agreement of proteomic and transcriptomic data.

The secretome of transplanted cells plays an important role in autocrine and paracrine signaling within the recipient tissue microenvironment. Even though several recent studies were focused on the secretome of NSCs, one may assume that the secretome will be significantly affected by cell origin and cultivation conditions; thus, results obtained by secretome analysis of Olig2-transduced human NSCs (Kim et al., 2014) or human NSCs derived from glioblastoma (Okawa et al., 2017) might not be fully transferable to other NSCs. Therefore, we analyzed the secretome of H9 NSCs during S and BG differentiation. Most of the 28 analyzed secreted growth factors, cytokines and chemokines, were detected at levels close to the lower limit of detection. However, consistently for all factors, the highest concentrations were secreted by proliferating NSCs. Eminent downregulation of secretion was detectable at the beginning of both differentiations (day 7), likely associated with abrupt microenvironment change after growth factor withdrawal. It is possible that levels of factors secreted by NSCs may be overestimated in our experiments due to the lower cell numbers in culture at the start of the differentiation (NSC condition) and subsequent normalization to the cellular protein concentrations. However, this normalization should not affect trends in differentiating cells. We detected increasing secretion of IL-6 and VEGF in both differentiations, but again there was growing difference between protocols over time, with cells grown in BG differentiation protocol secreting significantly more IL-6 and VEGF at day 28. IL-6 is known to stimulate proliferation and thus self-renewal of NSCs in murine brain (Storer et al., 2018). Another study provided evidence that murine NSCs do not express a functional receptor for IL-6 and do not secrete IL-6 at the detectable level. However, treatment of these NSCs with fusion protein consisting of IL-6 and its receptor resulted in NSC differentiation into neurons and astrocytes (Islam et al., 2008). Nevertheless, Oh et al. showed that IL-6 secreted by astrocytes stimulates differentiation of NPCs to neurons in rat brain (Oh et al., 2010). Secretion of IL-6 by human NSCs transduced with Olig2 suggests that IL-6 may also play a role in differentiation into oligodendrocytes (Kim et al., 2014). Interestingly, secreted IL-6 induces VEGF expression (Cohen et al., 1996). The effect of VEGF on NSCs will be discussed in detail below.

KEGG, GO, and Mfuzz analyses confirmed high similarity between S and BG differentiation and revealed downregulation of mRNA splicing and mitotic cytokinesis during both differentiations as expected for differentiating cells. On the other hand, neurogenesis, gliogenesis, synaptic vesicle cycle, Wnt pathway, fatty acid metabolism, and regulation of cytoskeleton organization were upregulated during both differentiations, and such processes may correspond to changes in cellular morphology and metabolism of differentiated cells (NPCs and neurons). The discrepancy in expression of proliferation markers Ki-67 and PCNA that we observed at the early stage of NSC differentiation is likely caused by additional functions of these proteins. Ki-67 plays roles in the regulation of the cell cycle, perichromosomal layer assembly during mitosis, or maintenance and localization of heterochromatin [reviewed in Sun and Kaufman (2018)]. PCNA plays essential roles in DNA replication and repair (Essers et al., 2005). This is in agreement with our results from Mfuzz clustering, which revealed that proteins with the highest abundance at day 7 of both differentiations are connected to DNA repair processes. Indeed, published results of gene expression analyses of abovementioned processes and pathways during NSC/NPC differentiation support our data, e.g., inhibition of proliferation, downregulation of RNA transcription, and activation of the Wnt pathway in differentiating cells (Gurok et al., 2004; Cai et al., 2006). Although several proteomic studies of neural differentiation from ESCs or NSCs exist, these studies were mainly focused on identification of novel differentiation markers [reviewed in Melo-Braga et al. (2015)]. Fathi et al. analyzed proteome changes during neural differentiation of human ESCs and observed an increased expression of proteins connected to transport (of vesicles, proteins, and neurotransmitters), redox homeostasis, and glycolysis in differentiated cells, whereas proteins connected to mRNA processing were decreased (Fathi et al., 2014). A study of proteome and phosphoproteome during differentiation of immortalized human NSCs showed downregulation of proteins involved in cell cycle and proliferation, while proteins involved in gliogenesis, neurogenesis, synaptogenesis, and Wnt signaling pathway were upregulated (Song et al., 2019). Wang et al. analyzed phosphoproteome during rat NSC differentiation and identified changes of phosphorylation status of 20 proteins participating in Wnt signaling (canonical and non-canonical pathways) and confirmed the Wnt signaling pathway as a key regulator of NSC differentiation (Wang S. et al., 2016). All these data agree with our results from KEGG and GO analyses.

Among the pathways identified as activated during both S and BG differentiations in our SWATH-MS data were the HIF-1 signaling pathway, Wnt signaling pathway, and VEGF signaling pathway. HIF1-α activates gene expression in response to hypoxia including gene for VEGF (Forsythe et al., 1996). However, our cultivations were performed in normoxic conditions, suggesting that the HIF1-α signaling pathway plays another role(s) in NSC differentiation. Recently, HIF1-α was shown to regulate neurogenesis by blocking premature differentiation of NSCs to neurons (Večera et al., 2020). Our immunoblotting results show decrease of HIF1-α abundance during both differentiations, which may correspond to increase in neuronal differentiation. Interestingly, expression of both HIF1-α and VEGF may be regulated by IL-6 via signal transducer and activator of transcription 3 (STAT3) (Loeffler et al., 2005; Xu et al., 2005). Moreover, activation of the Wnt signaling pathway in glioblastoma under normoxic conditions leads to an activation of the VEGF signaling pathway via HIF1-α signaling (Vallée et al., 2018). We observed a positive correlation between secretion of IL-6 and VEGF, as well as between abundance of catenin β-1 and VEGF secretion during both S and BG differentiations. Therefore, one can assume that these three signaling pathways—HIF-1, Wnt, and VEGF—play important and coordinated roles in NSC differentiation *in vitro*, which are independent on oxygen level.

As secretome analysis revealed elevated secretion of VEGF-A during both differentiation protocols, we wanted to know which VEGF-A isoform is secreted. Using immunoblotting, we detected increased abundance of VEGF121 during both NSC differentiations compared to NSCs. However, the highest expression of VEGF121 in cells was at the early stage of differentiations, whereas VEGF-A secretion was highest at the end of the differentiations. Surprisingly, we did not detect any protein expression of VEGF165, although this is the most expressed isoform in rat NSCs (Schänzer et al., 2004). We also detected increased abundance of VEGF-A receptor neuropilin-1 during both differentiations. Neuropilin-1 was initially identified as a receptor for VEGF165 (Soker et al., 1998) but later was shown to bind also VEGF189, but not VEGF121 in mouse neurons *in vivo* (Tillo et al., 2015). However, neuropilin-1 binds VEGF121 in human endothelial cells *in vitro* (Pan et al., 2007). Although expression of neuropilin-1 is induced by VEGF165 in rat NSCs (Maurer et al., 2003), our results suggest that increased abundance of neuropilin-1 during both differentiations may not be connected to VEGF165 expression and secretion in H9 NSCs.

VEGF165 is the most studied VEGF-A isoform. It was shown that VEGF165 stimulates proliferation of chicken retinal progenitor cells and inhibits their differentiation to retinal ganglion cells both *in vitro* and *in vivo* (Hashimoto et al., 2006), suppresses apoptosis and induces proliferation of rat NSCs in both the presence or absence of EGF/FGF2 *in vitro* (Schänzer et al., 2004), and supports survival of rat neurons *in vitro* (Hao and Rockwell, 2013). Moreover, supplementation with exogenous VEGF165 or transplantation of genetically engineered cells expressing VEGF165 provides neuroprotection and decreases histopathological changes in a rat model of brain ischemia (Manoonkitiwongsa et al., 2004; Yao et al., 2016), alleviates neuronal death in rat models of Huntington's (Ellison et al., 2013) and Parkinson's (Yasuhara et al., 2005) disease, and prolongs the lifespan of a mouse model of amyotrophic lateral sclerosis (Wang Y. et al., 2016). In contrast, we did not observe any effect of VEGF165 supplementation on proliferation and survival of NSCs, independently on the presence or absence of EGF/FGF2. This might be caused by a different regulation or sensitivity of the VEGF-A pathway in H9 NSCs or possibly by a too high dose of VEGF165 in our experiment. Several studies showed that VEGF165-induced proliferation and apoptosis inhibition are dose-dependent, i.e., higher concentration of VEGF165 (up to 100 ng/mL) has higher impact (Schänzer et al., 2004; Hashimoto et al., 2006), while other studies provided evidence that higher doses of VEGF165 (100 ng/mL and more) have lower or even opposite effects and may be neurotoxic (Manoonkitiwongsa et al., 2004; Yasuhara et al., 2005; Ellison et al., 2013).

We found that VEGF121 supplementation induced proliferation and enhanced the survival of H9 NSCs in the absence of other growth factors but had no additional effect in the presence of EGF/FGF2. Herrera et al. showed that VEGF-A expression is decreased in the spinal cord injury region for up to 1 month, but VEGF165 supplementation has no effect on neuronal survival. However, antibody inhibition of all VEGF-A isoforms led to an even lower number of surviving neurons, suggesting that other VEGF-A isoform(s) than VEGF165 may play a neuroprotective role (Herrera et al., 2009). Indeed, rat NSCs transfected with VEGF121 gene survived, migrated after transplantation to ischemic brain, and improved the Neurological Severity Scale score earlier when compared to control NSCs (Zhu et al., 2005). Furthermore, secreted VEGF121 is highly diffusible as it lacks the heparin-binding domain, VEGF165 contains one heparin-binding domain and thus is partially diffusible and partially bound to extracellular matrix, and VEGF189 with its two heparin-binding domains is tightly bound to the extracellular matrix (Park et al., 1993). Thus, VEGF121 secreted by transplanted cells or infused during medical treatment could possibly affect a larger region of tissue than the other two isoforms, which could be beneficial for therapy.

Based on the results of VEGF121 supplementation and increased abundance of VEGF121 during both differentiations, it is possible that VEGF121 is secreted during NSC differentiations to support survival of neuronal cells. In fact, we observed increased activation of caspase-3 (apoptotic marker) at the early stage of NSC differentiation, which can be assigned to a change of the microenvironment, but activation of Caspase-3 was decreased at a later stage of both differentiations, which may correlate with increased secretion of VEGF-A.

## Concluding Remarks

Previous studies showed that cell lines of the same cell type may differ in their gene expression and differentiation potential, thus making generalization of results complicated [reviewed in Melo-Braga et al. (2015)]. Although our results in global are supported by previously published data, we observed preferential differentiation of H9 NSCs into neurons, which is not typical for NSCs in general. This represents a limitation of the H9 NSC line as a model of true multipotent NSCs. However, NSCs derived from H9 ESCs are broadly used for both *in vitro* and *in vivo* (cell transplantations to animal models) studies related to CNS pathologies, including autism spectrum disorders (Nguyen et al., 2018), brain ischemia (Green et al., 2018), glioblastoma (Balbous et al., 2014), neuroblastoma (Carr-Wilkinson et al., 2018), or Parkinson's disease (Iacovitti et al., 2007). Moreover, protocol for isolation of the clinical grade NSCs from H9 ESCs was recently established (Bohaciakova et al., 2019). Thus, we believe that our comprehensive proteome and secretome analyses provide additional information applicable in future H9 NSC studies. In potential clinical settings, commitment into NPCs and neural lineage could be beneficial. In a pro-inflammatory, pro-glial microenvironment in spinal cord injury, leading to glial scar formation (Bradbury and Burnside, 2019), neurally committed cells might be a better option for supporting restoration of neuronal connections. A case study of tumorigenesis via glioneuronal tumor formation after NSC transplantation in human (Amariglio et al., 2009) shows yet another risk of multipotent stem cell transplantation, which could be potentially diminished by use of only neurally committed cells.

Overall, our results show large-scale proteome changes in human H9 NSCs differentiating *in vitro*, consistent with the program of committed neural differentiation. Key pathways identified as regulated during this process were VEGF, Wnt, and HIF-1 signaling pathways. We proved that VEGF121 induces proliferation and support survival of differentiating cells, which is accompanied by the increased expression of the VEGF-A receptor neuropilin-1, by the increased expression of regulators of VEGF expression IL-6 and catenin β-1 (and also HIF1-α in the early stages of NSC differentiation), and by the fluctuating levels of the apoptotic marker caspase-3. H9 NSCs on their own secreted increasing levels of IL-6 and VEGF-A over the differentiation time course. This secretory phenotype could be potentially beneficial as part of neuroprotective and modulatory effect of transplanted cells in cell-based therapies.

## Data Availability Statement

The datasets from the SWATH-MS measurements for this study can be found in the Panorama Public repository (https://panoramaweb.org/NSCsdifferentiation.url) and on ProteomeXchange under ID PXD021860.

## Author Contributions

JČ, JT, PV, and HK designed the study. JČ, JT, HK, KV, IP, IV, TP, and PV performed the experiments. JČ, JT, KV, HK, LP, MK, MV, and PV analyzed and interpreted the data. JČ, JT, HK, and PV wrote the paper with input from all authors. JČ, HK, and PV received the funding. All authors have seen and approved the manuscript.

## Conflict of Interest

The authors declare that the research was conducted in the absence of any commercial or financial relationships that could be construed as a potential conflict of interest.
