# Supplementary material for: Proteomic Characterization of Human Neural Stem Cells and Their Secretome During in vitro Differentiation
Source: Front Cell Neurosci. 2021 Jan 28;14:612560. doi: 10.3389/fncel.2020.612560 (PMC7876319; doi:10.3389/fncel.2020.612560)
Supplement: Supplementary file 1 [file Data_Sheet_1.docx]

Supplementary Figures


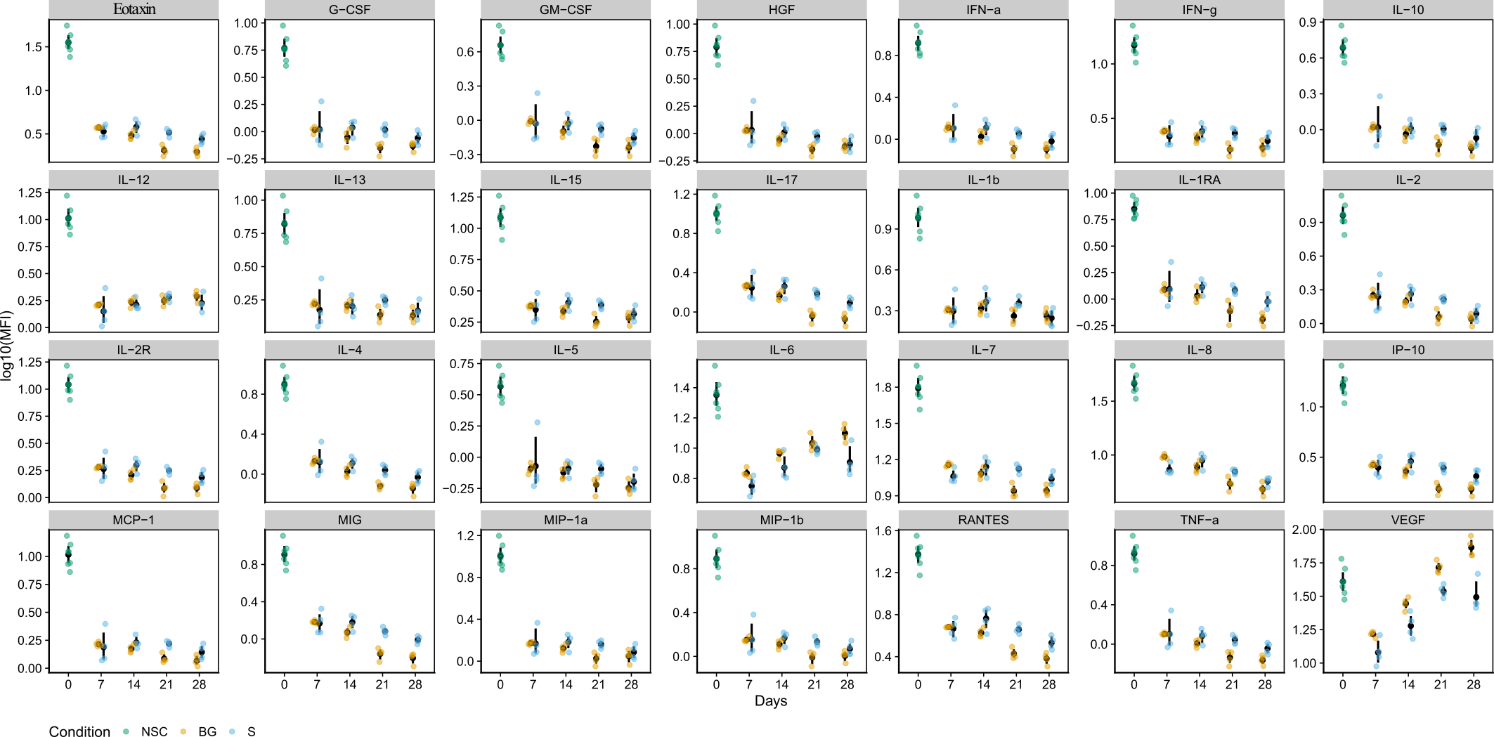


**Supplementary Figure 1.** **NSC secretome.** Graphs of the normalized log10(MFI) values for 28 secreted growth factors and cytokines in medium conditioned for two days in presence of H9 NSCs during the differentiation time course. Granulocyte colony-stimulating factor (G-CSF), Granulocyte-macrophage colony-stimulating factor (GM-CSF), Interferon α (IFN-α), Interferon γ (IFN-γ), Interleukins (IL-1β, IL-1RA, IL-2, IL-2R, IL-4, IL-5, IL-6, IL-7, IL-8, IL-10, IL-12 p40/p70, IL-13, IL-15, IL-17) and Tumor necrosis factor α (TNF-α); chemokines: Eotaxin, C-C motif chemokine 2 (MCP-1), C-C motif chemokine 3 (MIP-1α), C-C motif chemokine 4 (MIP-1β), C-C motif chemokine 5 (RANTES), C-X-C motif chemokine 9 (MIG) and C-X-C motif chemokine 10 (IP-10); growth factors: Hepatocyte growth factor (HGF) and Vascular endothelial growth factor (VEGF).

EGF and FGF2 were excluded from analysis as they were present in the medium used for preparation of standards.


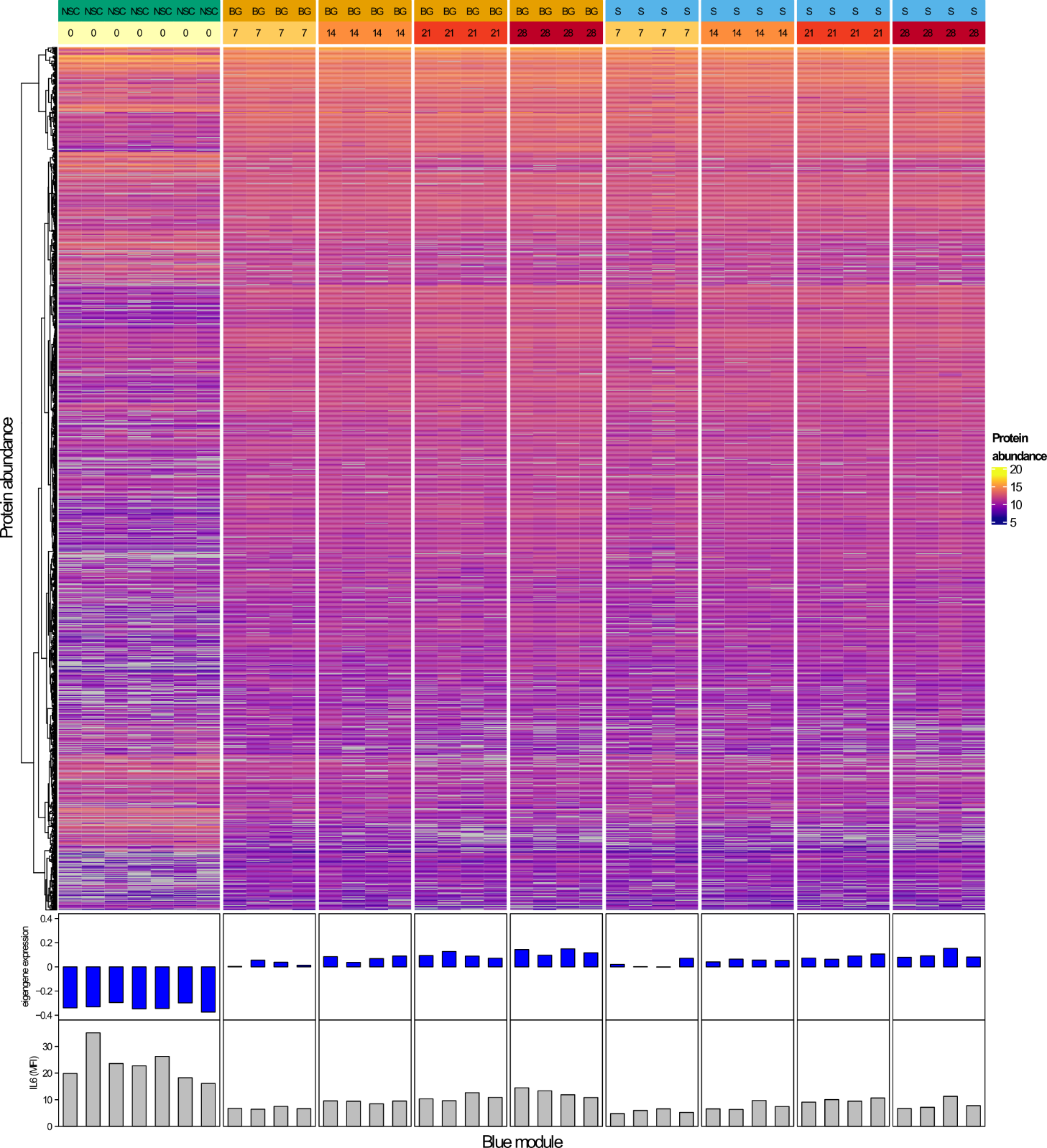


**Supplementary Figure 2.** **Protein co-expression network.** Heatmap of blue module protein abundance, together with module eigengene expression in individual samples and secreted IL-6 levels. Blue module consists mainly of proteins with low abundance at NSCs stage and increasing over time course of differentiation.


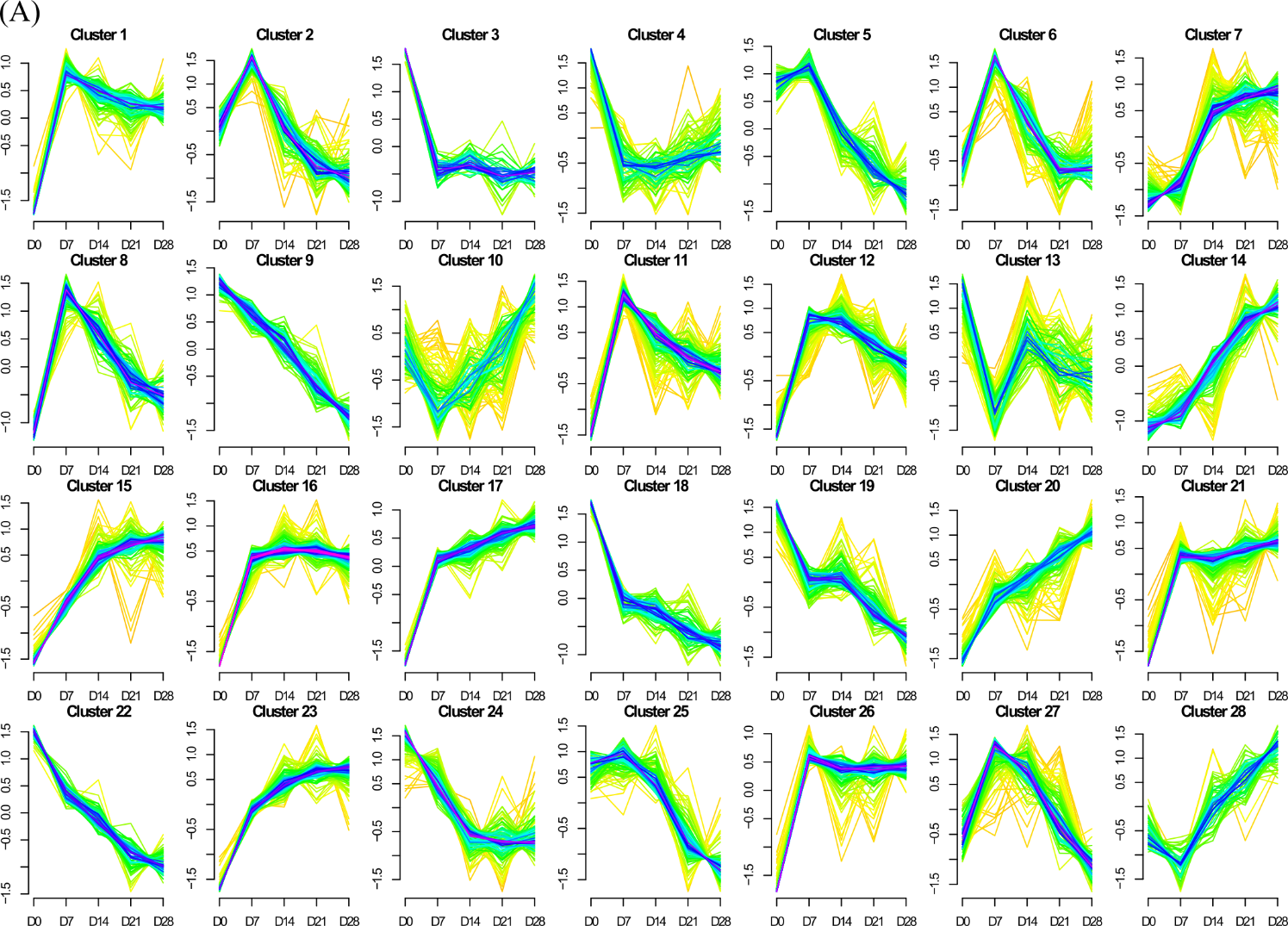


**
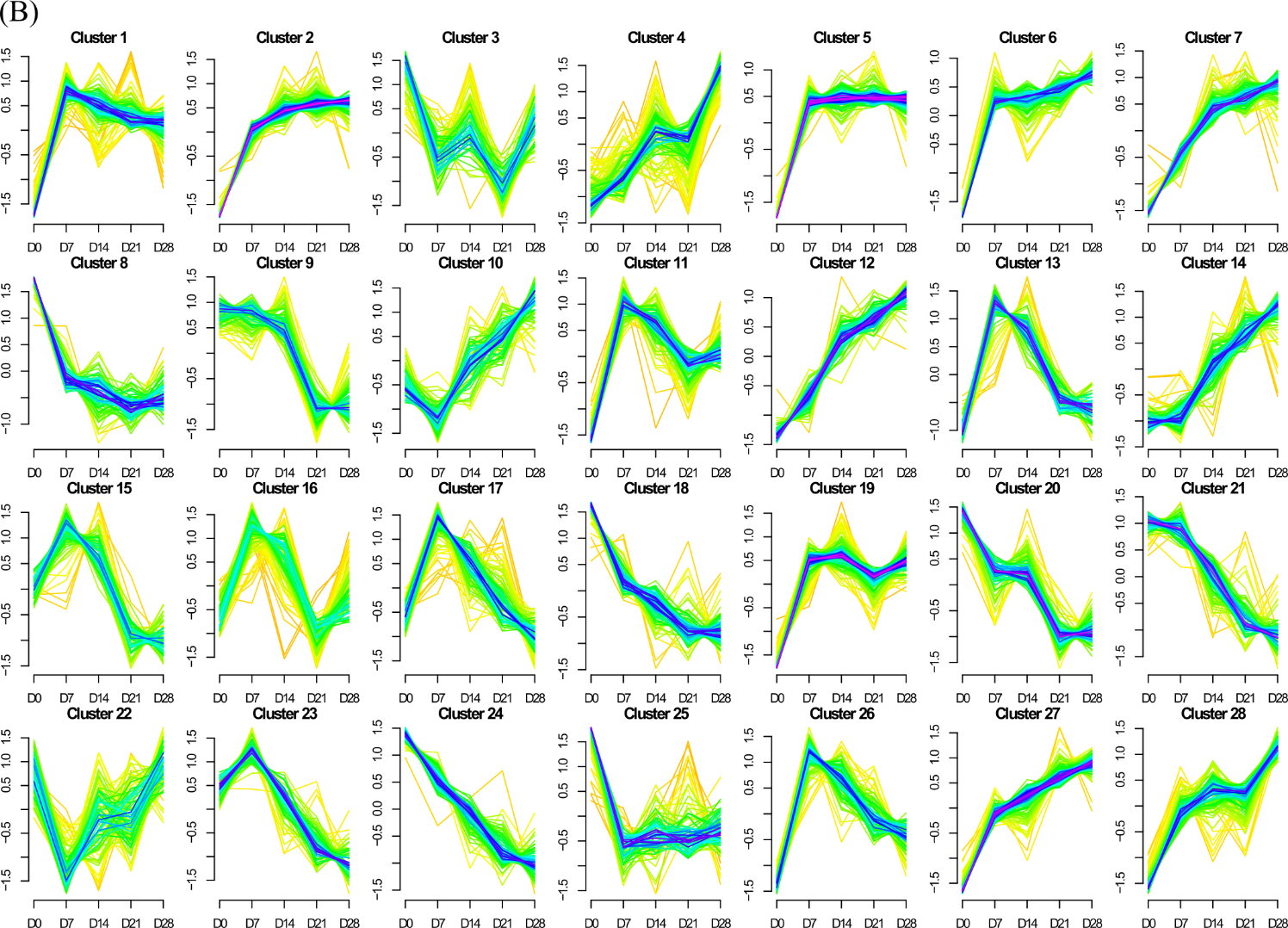
**

**Supplementary Figure 3.** **Mfuzz soft clustering of proteins into groups with similar temporal expression profile.** Proteins quantified in spontaneous (A) or BDNF/GDNF supported differentiation (B) were clustered separately, 28 clusters were obtained for each condition.


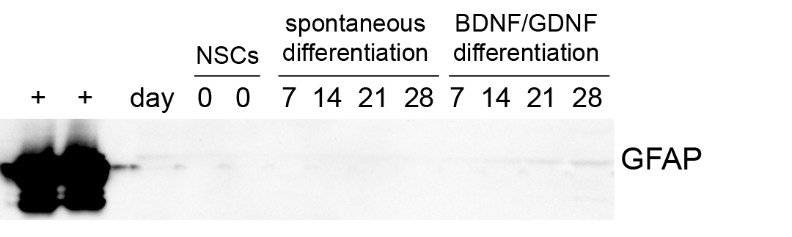


**Supplementary Figure 4. Detection of Glial fibrillary acidic protein (GFAP) by immunoblotting.** Positive controls (+) correspond to pig brain homogenate. Representative image from 2 biological replicates is shown.

Sample preparation:

Cells were lysed in ice-cold RIPA buffer (150 mM NaCl; 5 mM EDTA, pH 8; 50 mM Tris HCl, pH 7.4; 0.5% NP-40; 1% sodium deoxycholate; 1% Triton X-100 and 0.1% SDS) with 1x Halt protease and phosphatase inhibitor cocktail (Thermo Fisher Scientific). Porcine brain was obtained from control pig sacrificed in other experiments carried out at the institute, approved by the Resort Committee of the Czech Academy of Sciences, approval No. 53/2015. The brain tissue was homogenized in PBS on ice and proteins were extracted in RIPA buffer. Lysates were sonicated in an ice bath, and unlysed debris was pelleted by centrifugation for 10 min at 16,000 g at 4°C. Protein concentration in supernatants was determined by BCA assay (Thermo Fisher Scientific).

Three μg of proteins were separated in 3-8% Tris-Acetate gradient NuPAGE gels (Invitrogen) under reducing conditions according to the manufacturer’s instructions. Proteins were transferred by Trans-Blot Turbo transfer system (Bio-Rad) to the nitrocellulose membrane. Membrane was blocked 1 hour at room temperature and incubated overnight at 4°C with primary antibody (see Supplementary table 1B for details). Membrane was washed three times 10 min in Tris-buffered saline with 0.05% Tween 20 (TTBS) and incubated 60 min at room temperature with appropriate secondary antibody diluted 1:10,000 in 5% dry skim milk in TTBS. Membrane was washed in TTBS and incubated in ECL Prime reagent (Amersham). The Chemidoc XRS + detection system with Image Lab (version 5.2.1 build 11) software (Bio-Rad) were used to detect the chemiluminescent signal.
